# The formation and design of the 'Acute Admission Database'- a database including a prospective, observational cohort of 6279 patients triaged in the emergency department in a larger Danish hospital

**DOI:** 10.1186/1757-7241-20-29

**Published:** 2012-04-10

**Authors:** Charlotte Barfod, Marlene Mauson Pankoke Lauritzen, Jakob Klim Danker, György Sölétormos, Peter Anthony Berlac, Freddy Lippert, Lars Hyldborg Lundstrøm, Kristian Antonsen, Kai Henrik Wiborg Lange

**Affiliations:** 1 Department of Anaesthesia and Intensive Care, Hillerød Hospital, Hillerød, Denmark; 2 Department of Anaesthesia and Intensive Care, Aalborg Hospital, Aalborg, Denmark; 3 Department of Clinical Biochemistry, Hillerød Hospital, Hillerød, Denmark; 4 Deparment of Emergency Medicine, Hillerød Hospital, Hillerød, Denmark; 5 Emergency Medicine and Emergency Medical Services, Head Office, Capital Region of Denmark, Hillerød, Denmark

**Keywords:** Triage, Emergency Department, Database, Vital signs, Peripheral venous blood

## Abstract

**Background:**

Management and care of the acutely ill patient has improved over the last years due to introduction of systematic assessment and accelerated treatment protocols. We have, however, sparse knowledge of the association between patient status at admission to hospital and patient outcome. A likely explanation is the difficulty in retrieving all relevant information from one database. The objective of this article was 1) to describe the formation and design of the 'Acute Admission Database', and 2) to characterize the cohort included.

**Methods:**

All adult patients triaged at the Emergency Department at Hillerød Hospital and admitted either to the observationary unit or to a general ward in-hospital were prospectively included during a period of 22 weeks. The triage system used was a Danish adaptation of the Swedish triage system, ADAPT. Data from 3 different data sources was merged using a unique identifier, the Central Personal Registry number; 1) Data from patient admission; time and date, vital signs, presenting complaint and triage category, 2) Blood sample results taken at admission, including a venous acid-base status, and 3) Outcome measures, e.g. length of stay, admission to Intensive Care Unit, and mortality within 7 and 28 days after admission.

**Results:**

In primary triage, patients were categorized as red (4.4%), orange (25.2%), yellow (38.7%) and green (31.7%). Abnormal vital signs were present at admission in 25% of the patients, most often temperature (10.5%), saturation of peripheral oxygen (9.2%), Glasgow Coma Score (6.6%) and respiratory rate (4.8%). A venous acid-base status was obtained in 43% of all patients. The majority (78%) had a pH within the normal range (7.35-7.45), 15% had acidosis (pH < 7.35) and 7% had alkalosis (pH > 7.45). Median length of stay was 2 days (range 1-123). The proportion of patients admitted to Intensive Care Unit was 1.6% (95% CI 1.2-2.0), 1.8% (95% CI 1.5-2.2) died within 7 days, and 4.2% (95% CI 3.7-4.7) died within 28 days after admission.

**Conclusions:**

Despite challenges of data registration, we succeeded in creating a database of adequate size and data quality. Future studies will focus on the association between patient status at admission and patient outcome, e.g. admission to Intensive Care Unit or in-hospital mortality.

## Background

Systematic assessment and accelerated treatment protocols at Emergency Department (ED) admission have improved management and care of the acutely ill patient over the past years. Previous studies have mainly focused on specific diagnoses or prognostic values of isolated blood sample results, e.g. the effect of elevated concentrations of blood lactate in trauma [1], vital signs as a predictor of cardiac arrest [2] or intracerebral haemorrhage [3]. There is, however, only limited knowledge of how the initial assessment and status of the unselected acutely ill patient is associated with patient outcome. The limited clinical research in this field may partly be due to difficulties in retrieving all relevant and necessary information from one single database. Several registries exist, but they all focus on specific disease groups or interventions [4-6]. Some databases focus specifically on the emergency care process [7-9], but none of these include information on mortality and morbidity during or after ED admission.

The overall goal was to create a database allowing access to the following information:

• Patient status at admission, including vital signs, presenting complaint and triage category.

• Blood sample results, sampled at admission, including a venous acid-base (VAB) status.

• Outcome measures: length of stay (LOS), admission to Intensive Care Unit (ICU), in- hospital mortality, mortality 7 days and 28 days after admission, and discharge diagnosis.

Merging this information into a single database will enable us to analyze the potential associations between patient status at admission and defined outcome measures. To the best of our knowledge, a database of similar size and sufficient data quality has not been established previously. The objective of this article is to

1) describe the formation and design of the 'Acute Admission Database' and

2) characterize the cohort included.

## Methods

**Figure 1:**
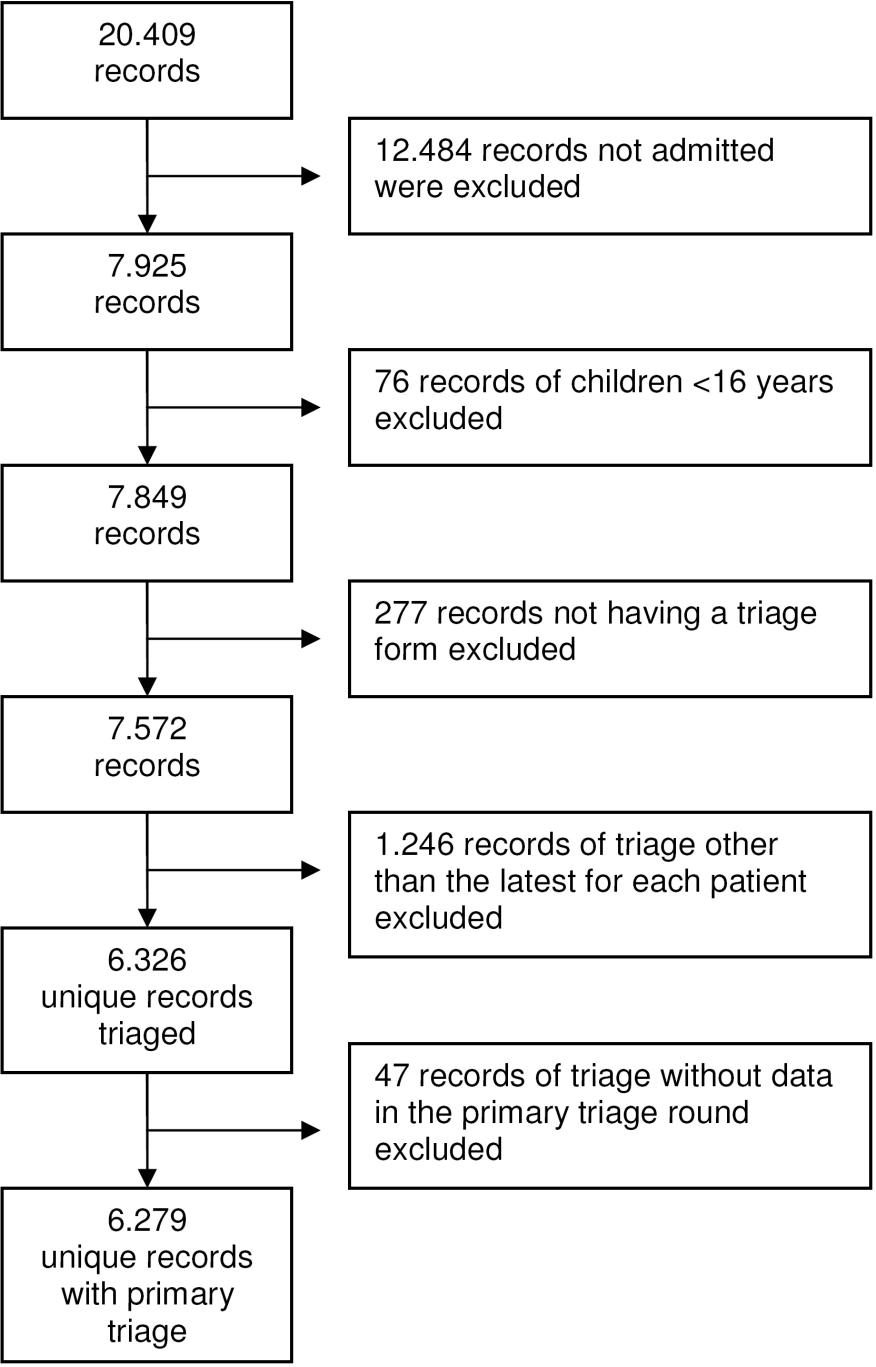
**Selection of the study cohort**. Patient contacts were excluded as explained in the figure. The final cohort included 6.279 patients, representing the latest admission for every patient triaged in the study period.

The 'Acute Admission Database' is a clinical database comprising data from acute admissions from the ED at Hillerød University Hospital, which is one of the largest specialized regional hospitals in the Capital Region of Denmark. The hospital is a 24-hour acute care hospital offering emergency, level-2 trauma, medical, surgical, and intensive care services for 310.000 citizens in North Zealand. The ED has approximately 50.000 patient contacts annually. We retrieved 6279 unique patients from the Acute Admission Database in the period from September 22, 2009 to February 28, 2010. Inclusion criteria were all patients aged > 16 years admitted from the ED, either to the ED observationary unit or to a general ward in-hospital. Patients with minor complaints and injuries that did not result in admission were excluded. Patients admitted more than once during the study period were only represented by the latest admission. Selection of the cohort is depicted in Figure 1.

### Data collection and quality

**Figure 2:**
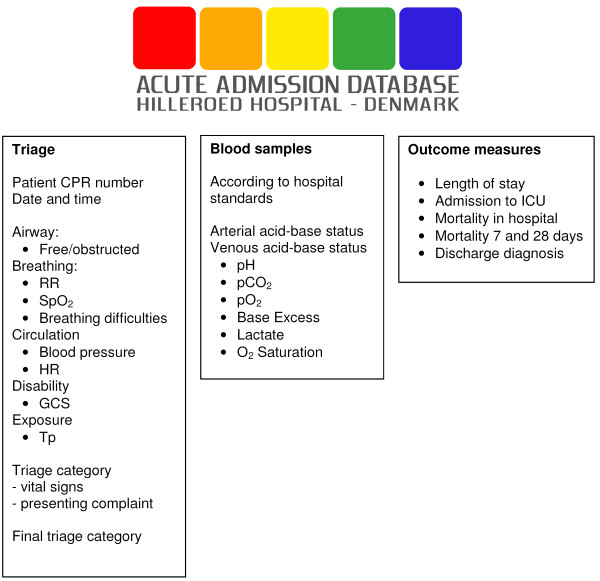
**Merging of three databases into the Acute Admission Database**. RR: respiratory rate; SpO_2_: saturation of peripheral oxygen (pulse oxymetry); HR: heart rate; GCS: Glasgow Coma Score; Tp: temperature; ICU: Intensive Care Unit; CPR: Central Personal Registry.

The data in the 'Acute Admission Database' derives from 3 sources (Figure 2):

1) Triage data: Data from initial assessment upon arrival, including time and date, vital signs, presenting complaint and triage category. Data was manually entered in the 'Acute Admission Database' as described below.

2) Blood sample results: Sampling performed at admission, including a venous acid-base (VAB) status retrieved from the hospital database, LABKA ll, version 1.4.2.H5 Computer Sciences Corporation (CSC).

3) Patient outcome: Data retrieved from the hospital database, OPUS Arbejdsplads, version 2.5.0.0 ^©^2010 Computer Sciences Corporation (CSC).

The three data sources were merged using the Central Personal Registry (CPR) number, which uniquely identifies gender and date of birth of all Danish citizens. The back-end database was designed in MSSQL and the front-end data-collecting tool was designed as a web application, enabling staff to report data directly from any workstation in the hospital using normal security validation. All data types were defined as Boolean, integer or numeric. The primary key was the unique CPR number. The secondary key consisted of the date and time of admission, making cross-reference possible with other health related databases. The final data set was of SQL type, making it suitable for export to almost any table format. We used IBM-SPSS Statistics v 18.0 (IBM Corporation) for data analysis.

#### Triage system and triage data

Hillerød hospital started using 'Hillerød Adaptive Process Triage' (HAPT) [10] in May 2009, having no previous experience with formalized triage. HAPT is inspired by the Swedish Adaptive Process Triage model, ADAPT [11], and has subsequently evolved into the 'Danish Emergency Process Triage' [12], which is currently under implementation at several hospitals across the country. The triage system ranks patients into five colour-coded triage categories. Each patient is assigned a triage level for each of the two main descriptors 1) vital signs and 2) presenting complaint. The variable of the two associated with the more urgent triage category determines the final colour-coded triage level, which in turn determines the level of patient monitoring, treatment and re-evaluation. The triage categories are 1) red (resuscitation, re-evaluation every 0 minutes (min)), 2) orange (emergent, re-evaluation every 10 min), 3) yellow (urgent, re-evaluation every 60 min), 4) green (non-urgent, re-evaluation every 180 min) and blue (minor injuries or complaints, re-evaluation every 240 min). Patients in blue triage category were not admitted and therefore not included in the Acute Admission Database. For further information about the details in the triage system, please consult references [10,12]. Data collected from the admission process was manually transferred from the triage form into the database. Predefined validation rules and filters controlled the quality of data entry. There were two validation rules for every manually entered numeric value: 1) An absolute range. Data outside this range was not allowed into the database. 2) An uncertainty range, requiring an extra validation from the user. The triage category for the presenting complaint was assigned according to a presenting complaint algorithm [10,12], defining the acuity of each complaint within 29 predefined main categories. If the presenting complaint algorithm did not adequately cover the patient's condition, the patient was scored as 'no adequate category' and the final triage category was determined solely by the patient's vital signs. If the presenting complaint was not scored by the triage nurse or physician, the medical students responsible for database entry were allowed to assign a category according to the nurse's notes in the triage form (from one of the 29 predefined categories). A three-week test period was used to familiarize the students with the database and to reinforce the nursing staff in recording triage data as completely as possible. During the project period, random samples were taken to ensure correct triage of the patients as well as correct data entry into the database. All triage documents were stored in hard copy in order to be able to retrieve original data if necessary. All data entered into the database was subsequently reviewed by one of two authors (CB and MPL) by comparing data on the triage sheet with the data entered in the database for each patient. The two reviewers did not participate in the primary data entry. Review of patient admission data was performed consecutively, and the reviewers were unaware of patient outcome at the time of registration.

#### Blood sample data

Blood sampling was performed by a team of medical students, who had a four-day course on procedure and technique before starting the project. They were instructed to minimize tourniquet time and to release the tourniquet as soon as the needle was within the vein. The blood sampling procedure followed a predefined sequence to minimize the risk of pre-analytical errors. The hospital standard procedures included a VAB for all patients in red, orange and yellow triage categories, and this was the final sample obtained from the cubital vein. Blood sampling was performed as soon as possible after patient admission and primary triage. All blood sample results were included in the database. An arterial blood gas was obtained and analyzed on physician request and staffs were instructed to use arterial blood gas results if in need of acute acid-base status.

#### Outcome measures

Predefined outcomes were retrieved from the hospitals computerized information system. LOS, admission to ICU, and mortality during admission and within 7 or 28 days after admission were registered. Furthermore the receiving ward and the discharge diagnosis were retrieved.

### Data safety

The Acute Admission Database was placed on a MS SQL Server 2005, maintained by the Department of Information Technology at Hillerød Hospital. Due to the private nature of the data, user interaction with the database was logged. Transaction logging was done every half hour. All medical students authorized to enter the database were obliged by duty of confidentiality. All had an extensive course in the use of the database and a personal log in. The two reviewers had extended rights and were the only ones with access to change data after logging in order to be able to correct errors in the process of data entry.

### Ethics

The study was approved by The Danish National Committee on Biomedical Research Ethics, J.nr. H-A-2009-006, and the Danish Data Protection Agency, Copenhagen, J.nr. HIH 2009-2 Akutdatabasen.

## Results

The characteristics of the cohort and the distribution within triage categories are shown in Table 1.

**Table 1:** Distribution of gender and age within the final triage category in the primary triage round

		Triage final				
		Red	Orange	Yellow	Green	Total
**Gender**						
	Male	156	778	1157	940	3031 (48.3)
	Female	122	806	1270	1050	3248 (51.7)

**Age(yr)**						
	< 45	71	291	583	575	1520 (24.2)
	45-54	33	181	323	263	800 (12.8)
	55-64	45	255	420	333	1053 (16.8)
	65-74	52	332	486	338	1208 (19.2)
	> 75	77	525	615	481	1698 (27.0)

**Total**		278 (4.4)	1584 (25.2)	2427 (38.7)	1990 (31.7)	6279 (100)

The figures in parentheses are percentages of total

**Figure 3:**
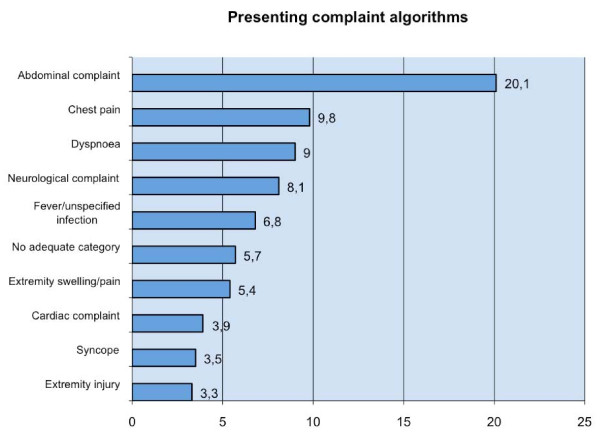
**The most common presenting complaint algorithms**. The presenting complaint at admission, assigned by the triage nurse or physician. Subgroups within each presenting complaint category are not shown.

The age distribution did not follow a Gaussian curve, but demonstrated a right skewed curve towards higher age. The distribution of vital signs in the primary triage round is shown in Table 2. Vital signs were within the defined normal range [10,13] in 75% of the patients, while 25% had one or more abnormal vital signs. The most common abnormal vital signs were temperature (tp) (10.3%), saturation of peripheral oxygen (SpO_2_) (9.2%), Glasgow Coma Score (GCS). (6.6%) and respiratory rate (RR) (4.8%). Missing variables in the primary triage round were most often tp (32.9%), RR (12.5%), GCS (5.2%) and score of the presenting complaint (6.0%). The most common presenting complaint algorithms are depicted in Figure 3.

**Table 2:** Vital signs in the primary triage round

	Range	N	%	missing (%)
**Total**		**6279**		**0 (0)**

SpO_2 _(%)	95-100	5509	87.7	191 (3.0)
	90-94	442	7.0	
	80-89	119	1.9	
	< 80	18	0.3	

RR (min^-1^)	> 35	38	0.6	785 (12.5)
	31-35	53	0.8	
	26-30	215	3.4	
	8-25	5187	82.6	
	< 8	1	0.0	

BP (mmHg)	90-	6101	97.2	120 (1.9)
	80-89	38	0.6	
	< 80	20	0.3	

HR (min^-1^)	> 130	120	1.9	126 (2.0)
	121-130	123	2.0	
	111-120	272	4.3	
	50-110	5570	88.7	
	40-49	57	0.9	
	< 40	11	0.2	

GCS	15	5537	88.2	327 (5.2)
	14	218	3.5	
	9-13	141	2.2	
	≤ 8	56	0.9	

Tp (°C)	> 40	16	0.3	2064 (32.9)
	38.1-40	371	5.9	
	36.1-38	3557	56.6	
	32-36	270	4.3	
	< 32	1	0.0	

SpO_2_: saturation of peripheral oxygen (pulse oxymetri); RR: respiratory rate; BP: systolic blood pressure; HR: heart rate; GCS: Glasgow Coma Score; Tp: temperature

In 5.7% of the patients, no adequate presenting complaint algorithm was found. VAB status was taken in 2674 patients, corresponding to 43% of all patients and to 53% of the patients in the red, orange and yellow triage categories. Results, definitions and grouping of acid-base disturbances are shown in Table 3. pH was within normal limits (between 7.35 and 7.45) in 78% of the patients, who had a VAB taken, while 15% had a pH less than 7.35 (acidosis) and 7% had a pH above 7.45 (alkalosis). Acidosis was caused by elevated partial pressure of carbon dioxide (pCO_2_) (respiratory acidosis) in 77% of the patients, while 25% had metabolic acidosis. Within the group of patients with a normal pH, approximately half of the patients had normal blood gas values, while the remainder had a compensated normal pH in spite of an abnormal base excess (BE) or pCO_2_. In patients with alkalosis, 42% demonstrated a metabolic alkalosis and 22% a respiratory alkalosis.

**Table 3:** Acid-base status evaluated with a venous blood sample at admission

pH	N (%)	Acid-base status	N (%)
< 7.35	405 (15)	Respiratory acidosis^1^	311 (77)
		
		Metabolic acidosis^2^	100 (25)
		
		Mixed respiratory/metabolic acidosis^3^	10 (2.5)
		
		Acidosis other^4^	16 (4)

7.35-7.45	2069 (77)	Normal acid-base status^5^	976 (47)
		
		Compensated^6^	1093 (53)

> 7.45	200 (8)	Respiratory alkalosis^7^	44 (22)
		
		Metabolic alkalosis^8^	83 (42)
		
		Mixed respiratory/metabolic alkalosis^9^	1 (0.5)
		
		Alkalosis other^10^	73 (37)

^1 ^pH < 7.35 and pCO_2 _> 6

^2 ^pH < 7.35 and BE < -3

^3 ^pH < 7.35, pCO_2 _> 6 and BE < -3

^4 ^pH < 7.35, pCO_2 _< 6 and BE > -3

^5 ^7.35 ≤ pH ≤ 7.45, 4 ≤ pCO_2 _*≤ *6 and -3 ≤ BE ≤ 3

^6 ^7.35 ≤ pH ≤ 7.45, (pCO_2 _< 4 or > 6) or (BE < -3 or > 3)

^7 ^pH > 7.45 and pCO_2 _< 4

^8 ^pH > 7.45 and BE > 3

^9 ^pH > 7.45, pCO_2 _< 4 and BE > 3

^10 ^pH > 7.45, pCO_2 _> 4 and BE < 3

BE: Base Excess. Units for pCO_2 _are kPa

Within the pH group < 7.35 and > 7.45 the sum exceeds 100% due to patients represented in more than one group

The table illustrates complete cases. VBG results were missing for 3605 patients (57%)

Median LOS was 2 days with a minimum of 1 day and a maximum of 123 days. The distribution was heavily skewed to the left, as 75% of the patients had a LOS of 4 days or less, and 95% of the patients had a LOS of 12 days or less. The proportion of patients admitted to ICU was 1.6% (95% CI 1.2-2.0), 1.8% of all patients (95% CI 1.5-2.2) died within 7 days of admission and 4.2% (95% CI 3.7-4.7) died within 28 days of admission.

## Discussion

The main objective of the present study was to create a database from which data could easily be retrieved. Furthermore, the data should cover the entire process from admission to discharge or death. This seemingly simple task turned out to be much more challenging than expected.

The manual handling of data in the triage phase proved to be a major operational limitation of the 'Acute Admission Database'. The manual procedure by which the triage nurse registered and transferred presenting complaints and vital signs to the triage form resulted in missing data. Primary triage was performed either partly or fully in 96% of the patients. Not all vital signs were, however, recorded satisfactorily. Tp measurement was missing in 32.9%, possibly because tp was not a mandatory component of triage at the time of the project. RR and GCS were missing in 12.5% and 5.2%, respectively, of the primary triage rounds. A possible explanation may be that the recording of these variables involves a personal judgement, whereas BP and HR measurements are recorded 'automatically'. A patient may qualify for more than one presenting complaint algorithm, e.g. present with both 'chest pain' and 'dyspnoea'. Some patients (5.6%) did not fit into any algorithm, suggesting a need for a more comprehensive list of presenting complaints, e.g. like in the Swedish triage system ADAPT [11,14] or the Canadian Emergency Department Triage and Acuity Scale [15]. Also, in the procedure of blood sampling, missing data was a limitation. VAB results were only available in 53% of the patients in the red, orange and yellow triage categories. This could be due to technical problems in both sampling and analysis. Whether the missing VAB's were randomly distributed or will lead to any bias in the interpretation of the data will be discussed in a future article about the VAB results and their association with outcome. The data entry from the triage form to the database was done manually by a team of medical students, who were employed for the project period only, and therefore not a permanent solution. Finally, the review process of all data comparing the triage form to the data entered in the database was very time consuming.

The question is then; how do we proceed in creating a database with close to 100% data capture? This would require ongoing control of the completeness and quality of data as well as continuous education of the personnel. Furthermore, it is important to minimize the time spent on registration and, if possible, to do the registration bedside. The manual handling of data is very cumbersome and also poses the risk of error during data entry. An alternative to manual data entry is automatic data capture, where all vital signs, triage colours etc. are transferred automatically to a database, similar to the 'Swedish Emergency Registry'[9], a national database incorporating data from 6 ED's in Sweden. Automatic data capture solves the problem of data entry, but does not necessarily make data more valid or reliable. Furthermore it might require development of software that is not readily available.

### Internal and external data validity

The data validity relates to two aspects; first of all we wish to achieve data of high quality and coverage to be able to monitor variations in our activity and effect of diagnostic work up within the setup of our own hospital and region. Furthermore the results from the Acute Admission Database might be of interest to other hospitals with a similar profile; i.e. large hospitals with a broad intake of acute medical and surgical patients. According to the triage system, all trauma patients are assigned to the red triage category in the primary triage round, due to the presenting complaint, irrespective of vital signs. In our region, multi-trauma and suspected intracranial or intrathoracic injuries are directly referred to a level 1 trauma centre in Copenhagen without being admitted to Hillerød Hospital. The trauma patients in our cohort are therefore less ill but still fulfilling the criteria for trauma, which may affect the external validity of our data. With respect to the acute medical and surgical conditions, the population in the 'Acute Admission Database' is comparable to the populations in other larger acute hospitals in the country and probably in most of Scandinavia. Patients with minor complaints (blue category) were not included in the 'Acute Admission Database'. It is therefore not possible to analyze the patients with minor injuries and complaints and patients who left the ED without being seen.

### Data reliability

Extensive training of the staff was done prior to implementation of the triage system in May 2009 and internal audits were performed throughout the study period. Inter-rater reliability is notoriously a problem in systems partly based on subjective interpretation, and further studies are needed to assess inter- and intra-rater variability in the triage system used.

### Strength and future studies

In spite of the challenges discussed above, we succeeded in collecting data from a broad, unselected group of acutely ill, surgical and non-surgical patients, irrespective of diagnoses. An extensive review of the literature by Farrokhia et al. [16] found very few quality studies investigating the association between specific vital signs or reasons for the ED visit and mortality in the general acute population. The data collected in the 'Acute Admission Database' provides us with the opportunity to examine this question in more detail in future studies. Retrieval of blood sample results from the database will also allow us to incorporate laboratory results, especially venous acid-base status, into a model predicting adverse outcome in the acutely ill patient. The close association between arterial and peripheral venous blood lactate, pH and BE has been demonstrated in several studies [13,17,18], suggesting that venous blood gas sampling should be encouraged, especially in patients with metabolic disturbances.

## Conclusion

The assembly of data in the 'Acute Admission Database' proved to be both challenging and cumbersome. Despite the limitations discussed above, we succeeded in creating a database of adequate size and data quality. These data can easily be retrieved for further analysis of the association between patient status at admission to hospital and defined endpoints.

## Abbreviations

ADAPT: Adaptive Process Triage; BE: Base Excess; BP: Blood pressure; ED: Emergency Department; GCS: Glasgow Coma Score; HAPT: Hillerød Adaptive Process Triage; ICU: Intensive Care Unit; LOS: length of stay; min: minutes; pCO_2_: partial pressure of carbon dioxide; RR: respiratory rate; SpO_2_: saturation of peripheral oxygen (pulse oxymetry); Tp: temperature; CPR: Central Personal Registry.

## Competing interests

The authors declare that they have no competing interests.

## Authors' contributions

CB participated in the conception and design of the database, the present study, data collection and interpretation, drafted and critically revised the manuscript. KL, ML and JD participated in the conception and design of the database, the present study, data collection and interpretation and critically revised the manuscript. PB, GS and KA participated in the conception and design of the database, data collection and interpretation and critically revised the manuscript. LH and FL participated in data interpretation and critically revised the manuscript. All authors read and approved the final version of the manuscript
